# The expression of genes contributing to pancreatic adenocarcinoma progression is influenced by the respective environment

**DOI:** 10.18632/genesandcancer.173

**Published:** 2018-03

**Authors:** Micah N. Sagini, Michael Zepp, Frank Bergmann, Matthias Bozza, Richard Harbottle, Martin R. Berger

**Affiliations:** ^1^ Toxicology and Chemotherapy Unit, German Cancer Research Center (DKFZ), Heidelberg, Germany; ^2^ University Clinic of Heidelberg, Institute of Pathology, Heidelberg, Germany; ^3^ DNA Vectors, German Cancer Research Center (DKFZ), Heidelberg, Germany

**Keywords:** PDAC, gene expression profiles, tumor model, tumor micro-environment, TGM2

## Abstract

Pancreatic adenocarcinoma is a highly aggressive malignancy with dismal prognosis and limited curative options. We investigated the influence of organ environments on gene expression in RNU rats by orthotopic and intraportal infusion of Suit2-007^luc^ cells into the pancreas, liver and lung respectively. Tumor tissues from these sites were analyzed by chip array and histopathology. Generated data was analyzed by Chipster and Ingenuity Pathway Analysis (±1.5 expression fold change and p<0.05). Further analysis of functional annotations derived from IPA, was based on selected genes with significant modulation of expression. Comparison of groups was performed by creating ratios from the mean expression values derived from pancreas and respective *in vitro* values, whereas those from liver and lung were related to pancreas, respectively. Genes of interest from three functional annotations for respective organs were identified by exclusion-overlap analyses. From the resulting six genes, transglutaminase2 (TGM2) was further investigated by various assays. Its knockdown with siRNA induced dose dependent inhibitory and stimulatory effects on cell proliferation and cell migration, respectively. DNA fragmentation indicated apoptotic cell death in response to TGM2 knockdown. Cell cycle analysis by FACS showed that TGM2 knockdown induced G1/S blockade. Therefore, TGM2 and its associated genes may be promising therapeutic targets.

## INTRODUCTION

Pancreatic ductal adenocarcinoma (PDAC) is a lethal disease with a five year survival rate of less than 5% [1]. In the last three decades, a worrying trend of increased cases of PDAC has been witnessed in developed nations, resulting in high death toll [2][3][4][5]. The growth of tumor cells at sites distinct and distant from the primary organ is the underlying cause of cancer-related mortalities attributed to ∼90% of cancer deaths [6][7]. In pancreatic cancer, the seeding of tumor cells into distant organs has been shown to have a selective propensity to the liver, followed by the peritoneum, lungs, bones, and adrenal glands [8][9].

Metastasis per se, is not a solitary event; rather, a complex cascade involving the interplay of tumor and non-tumor cellular, as well as extracellular components within the tumor microenvironment. During this process, tumor cells are required to surmount and colonize an intact tissue, composed of different cell types, tight junction proteins and cell adhesion molecules (β1 integrin and E-cadherin), involved in myriad signaling pathways within the tumor microenvironment [10][11][12][13]. In pancreatic ductal adenocarcinoma, a dense desmoplastic reaction constituting up to 80% of the tumor volume, harboring ECM and numerous cellular components (immune cells, nerve cells, fibroblasts, stellate cells, growth factors and cytokines), has been well characterized [14][15][16][17].

Interactions of these components are mediated by a plethora of cellular proteins within the tumor microenvironment, which orchestrate tumor progression through cell migration and invasion processes [18][19]. Some of the key proteins involved in various signaling events within the stroma include, but are not limited to tumor growth factor(TGF), cadherin, matrix metalloproteinases(MMPs), integrin, β-catenin, tumor necrosis factor alfa (TNFα), c-MYC protein and scaffold proteins like transglutaminase2(TGM2) [20][6][21][22][23].

One of the underlying factors that influence tumor growth and progression is the interplay between tumor cells and stromal components [24]. Nevertheless, it is not entirely clear how the tumor environment contributes to PDAC progression [25]. It is however believed that perturbation of the epithelium, particularly during malignancy is bound to alter the tumor environment since it harbors both the extracellular matrix and cellular components, which are crucial in maintaining the integrity of epithelial tissues [26]. With increasing number of newly detected cases that correlate with the death toll, a profound understanding of factors governing metastatic disease is a central prerequisite for appropriate therapeutic modalities [27][28]. We hypothesized that tumor cells growing in different organ environments are subject to modulation of expression in regard to tissue architecture of the respective organ.

As a follow up on this question, human PDAC Suit2-007^luc^ cells were orthotopically as well as intraportally infused into pancreas, liver and lungs of RNU rats. The study was based on our prototype model [29], which was modified to include primary (pancreas) and metastatic (liver and lungs) organ sites. Suit2-007^luc^ PDAC cells growing in these organs were re-isolated and examined for changes in gene expression. The modulation of gene expression was analyzed to identify and profile genes that contribute to disease progression *in vivo*.

## RESULTS

**Figure 1:**
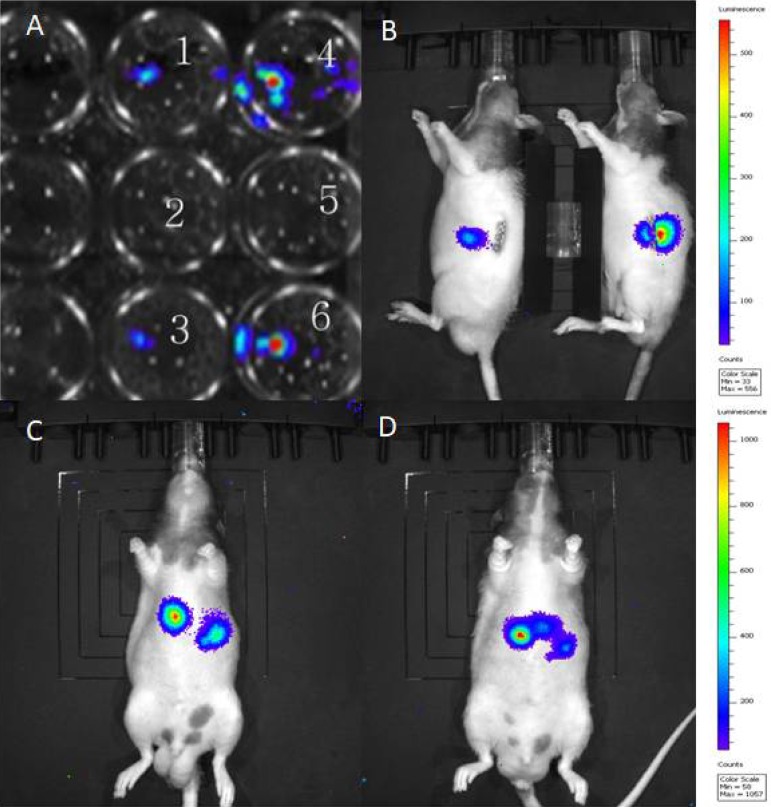
Light emission from luciferase transfected Suit-2007 cells **A** represents Suit-2007 cells (wild type) in wells number 2 and 5 (2.5 × 10^5^) and Luciferase transfected Suit-2007 cells, in wells number 1 and 3 (1.5 × 10^5^), 4 and 6 (2.5 x10^5^), which were incubated with luciferin for detection of light emission. **B** represents RNU rats bearing tumor following orthotopic implantation of Suit-2007 into the pancreas. In **C** and **D**, RNU rats were intraportally inoculated with Suit-2007^luc^ and tested for light emission after 7 days following intraperitoneal injection of luciferin.

**Figure 2:**
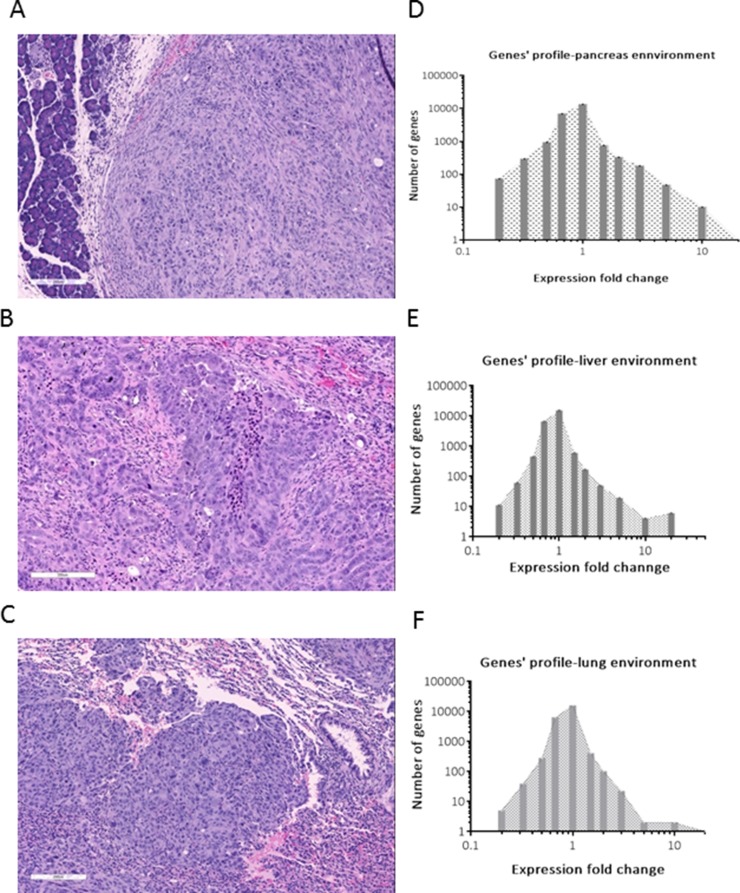
Characteristics of the rat model **A**, **B** and **C** represent H and E stained sections for pancreas (A), liver (B) and lung (C), respectively. The numerical analyses of gene expression for respective organs are shown **D**, **E** and **F** respectively. Expression fold change (X-axis) for pancreas (D) was obtained by using the *in vitro* expression levels as the denominator. In case of liver and lung (E and F), the expression levels in pancreas were used as the denominator.

The transfection of Suit2-007 cells with luciferase reporter gene for animal experiments was performed as described in materials and methods. Figure 1A represents transfected Suit2-007cells in a 24 well plate, showing light emission (wells number 1, 4, 3 and 6) following chemiluminescence testing. The wild type cells, which served as control, are also indicated (wells number 2 and 5). Figure 1B represents images of RNU rats with established tumor, following orthotopic implantation of Suit2-007 cells into the pancreas. Additionally, 1C and D, represent RNU rats with tumors following intraportal inoculation of Suit2-007^luc^ cells via the mesocolic vein. The rat model was intended to mimic orthotopic and metastatic disease for evaluation of genes associated with tumor progression. Orthotopic implantation resulted in the formation of solid tumor, consisting of atypical epithelial cells with polymorphous nuclei with frequent prominent nucleoli (Figure 2A). Intraportal infusion resulted in liver and lung metastases (Figure 2B and 2C).

The tumor tissues derived from these three sites (pancreas, liver and lung) were analyzed for histopathology by hematoxylin and eosin (H and E) method, as well as by chip array regarding the modulation of gene expression (Figures 2C, 2D and 2E).

From the tumor tissue originating from pancreas, 2662 genes showed a significant (> ±1.5 fold) up or down-regulation of expression when compared to control cells growing *in vitro*. Although the number of up or down-modulated genes was almost identical, the graphical representation (Figure 2D) was asymmetrical because few genes (*n* = 58) showed > 5 fold up-regulation. This pattern was replicated in genes from cells, which had grown in liver and lung tissues (Figures 2E and 2F). Interestingly, from all significantly modulated genes, 62% were up-regulated and 38% down-regulated in both organs of metastasis.

Gene profiling by Ingenuity Pathway Analysis (IPA) was performed with 2662 genes filtered from a total of 23,000 genes for having a significant (±1.5) expression fold change. With the IPA platform, various gene clusters were assigned to biological functions based on a significant Z-score (±2). The Z-score indicates activation or deactivation of a given gene cluster by comparing the observed (experimental) *versus* the expected values. When the observed and expected values match, the overall score has a positive value and vice versa. A summary of all identified functional annotations for pancreas, liver and lung is shown in Table 1.

**Table 1:** Functional annotations of Suit2-007 PDAC cells, re-isolated from pancreas, liver and lung

Functional annotations, pancreas environment^a^	*p*-Value^b^	Predicted activation^c^	Activation z-score^d^	Number of genes^e^
Cell movement	1,56E-11	increased	2,199	(178/326)
Cell signaling & interaction (interaction of tumor cells)	2,40E-10	increased	2,559	(60/109).
Gene expression	6,19E-09	increased	2,572	(143/341)
Cellular assembly, DNA replication, recombination & repair (nuclear foci formation)	3,33E-05	increased	3,142	(6/24)
Cell death & survival (cell death of squamous cell carcinoma)	4,18E-05	increased	2,414	(12/24)
Cell death & survival (viability of ovarian cancer cells)	1,69E-04	increased	2,215	(7/17)
Cancer, organismal injury & abnormalities	2,18E-07	decreased	−2,198	(45/59)
Infectious diseases	1,44E-12	decreased	−2,198	(156/362)
Cellular assembly, DNA replication, recombination, and repair (chromosomes alignment)	6,68E-06	decreased	−2,976	(8/12)
Cell cycle (M phase of tumor cell lines)	6,89E-05	decreased	−2,240	(14/29)
Cell cycle (progression of cervical cancer cells)	6,93E-05	decreased	−2,138	(9/29)
DNA replication, recombination, and repair	9,29E-05	decreased	−2,135	(11/30)
Cell cycle (interphase of cancer cells)	9,50E-05	decreased	−2,343	(18/20)
Cellular development	1,05E-04	decreased	−2,678	(6/10)
Cell cycle (re-entry into interphase)	1,09E-04	decreased	−2,121	(15/36)
**Functional annotations, liver environment^a^**				
Cell signaling & interaction (interaction of tumor cells)	1,23E-13	increased	2,191	(28/77)
Cellular movement, immune cell trafficking (leukocyte migration)	3,58E-05	increased	3,039	(23/49)
Inflammatory response	5,19E-06	increased	2,423	(25/49)
Cell signaling & interaction (activation of tumor cells)	3,91E-05	increased	2,011	(17/53)
Cellular movement (homing of cells)	1,84E-04	increased	2,998	(23/48)
Cellular growth & proliferation (colony formation)	5,90E-04	increased	2,044	(4/15)
Neurological, skeletal & muscular disorders (neuromuscular disease)	5,54E-05	increased	2,449	(48/106)
Neurological disease (progressive motor neuropathy)	3,02E-07	increased	2,449	(36/81)
Cell death & survival (cell death)	3,68E-16	decreased	−2,162	(103/306)
**Functional annotations, lung environment^a^**				
Cellular growth, development, &proliferation	1,06E-13	increased	2,979	(19/157)
Cell death & survival (tumor cells viability)	6,69E-07	increased	2,657	(29/77)
Organismal development	1,80E-04	increased	2,461	(19/19)
Gene expression (transactivation)	8,91E-07	increased	2,607	(20/46)
Infectious diseases (viral infection)	1,40E-04	increased	2,953	(41/116)
Lipid metabolism & molecular transport	1,54E-03	decreased	−2,176	(9/9)
Cellular movement (invasion of tumor cells)	1,03E-03	decreased	−2,157	(10/10)

a Functional annotations represent predicted biological functions of gene clusters derived from Ingenuity Pathway Analysis

b *p*-Value is the calculated probability derived from individual gene cluster in a defined annotation for the respective gene group.

c Predicted activation is the overall activation state (increased or decreased) of the functional annotation for respective genes in a given gene cluster

d Activation Z-score is a defined quantity, which determines whether a biological function has significantly more “increased” predictions than “decreased” predictions (Z > 0) or *vice versa* (Z < 0).

e Number of genes represent selected genes based on significant modulation of expression *versus* the total number of genes for respective functional annotation

For each annotation, the p-value, Z-score, predicted activation and number of genes respectively, is shown. In the pancreas, 15 functional annotations showed predicted activation or deactivation as indicated by a significant Z-score of greater or less than 2. Based on the respective p-values, infectious diseases, cell movement and cell signaling were most significantly modulated. In the liver environment, nine functional annotations were identified for their significant Z-score. From these, cell death and survival, as well as cell signaling were associated with the most significant p-values for modulation of gene expression. In the lung environment, seven functional annotations showed significant Z-scores, of which cellular growth, cell development and cell proliferation had the most significant p-values. The selection of genes belonging to three functional annotations for subsequent analysis was based on their consistent occurrence in all three tissues, exclusion of overlapping genes and their significant p-values in relation to cancer progression.

**Figure 3:**
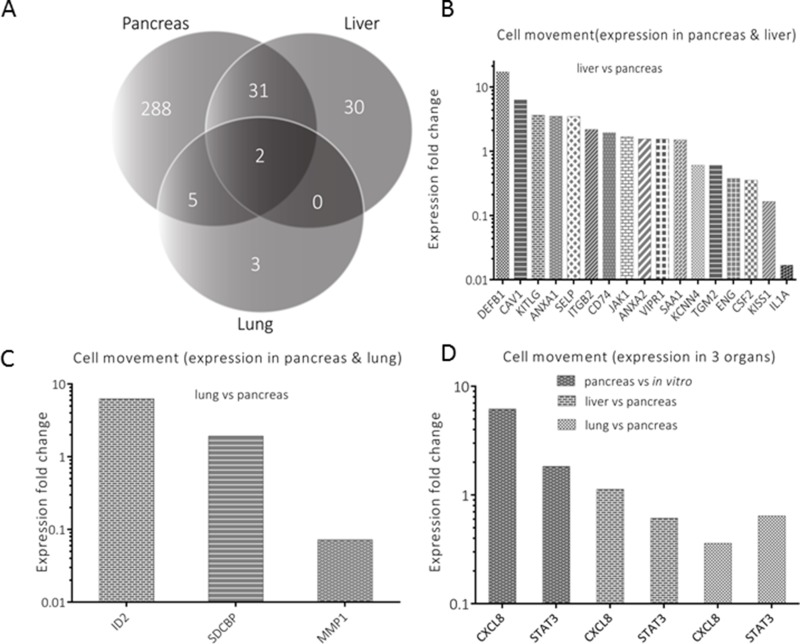
Analysis of genes annotated for cell movement **A** represents genes’ distribution (*n* = 359) for an exclusion-overlap analysis (Venn diagram) of cell movement, which were significantly modulated in the three organs. Thirty one (*n* = 31) genes were listed in pancreas and liver, five genes in pancreas and lung and two genes in all three organs. Significantly modulated genes are profiled in **B** (liver *versus* pancreas), **C** (lung *versus* pancreas) and D (expression in all organs), respectively.

**Figure 4:**
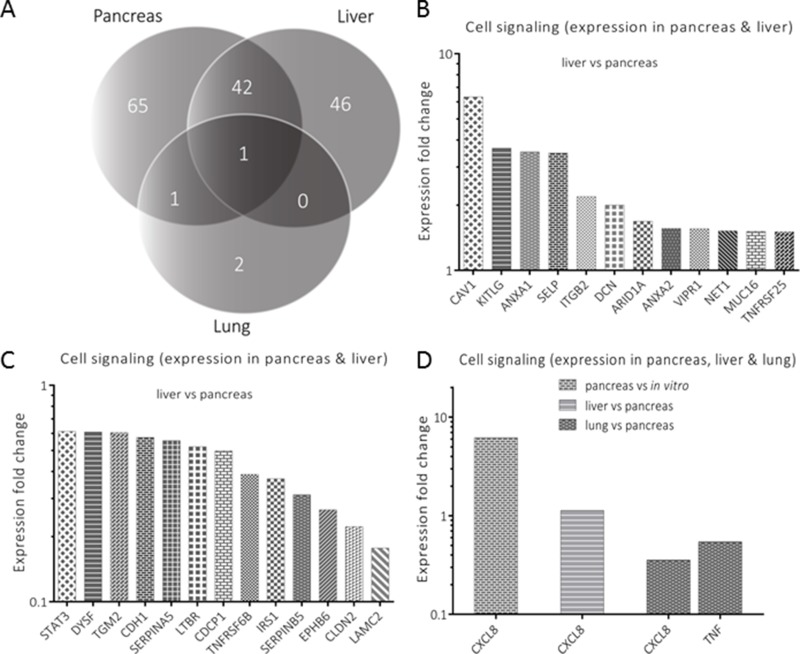
Analysis of genes annotated for cell signaling **A** represents genes’ distribution (*n* = 157) for an exclusion-overlap analysis (Venn diagram) of cell signaling, which were significantly modulated in the three organs. Forty two (*n* = 42) genes were listed in pancreas and liver, a single gene in pancreas and lung and another single gene in all three organs. Significantly modulated genes are profiled in **B** (liver *versus* pancreas), **C** (lung *versus* pancreas) and **D** (expression in all organs), respectively.

**Figure 5:**
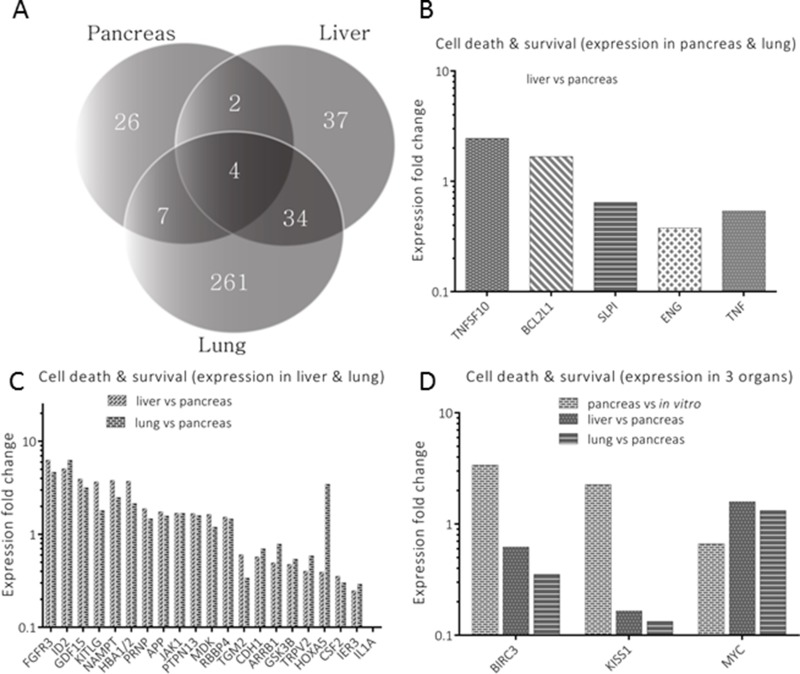
Analysis of genes annotated for cell death and survival **A** represents genes’ distribution (*n* = 371) for an exclusion-overlap analysis (Venn diagram) of cell death and survival, which were significantly modulated in the three organs. Two genes (*n* = 2) were listed in pancreas and liver, thirty four genes in liver, seven genes in pancreas and lung and four genes in all three organs. Significantly modulated genes are profiled in **B** (liver *versus* lung pancreas), **C** (lung *versus* pancreas) and **D** (expression in all organs), respectively.

The functional annotations considered as most important were further analyzed by exclusion-overlap analyses (Venn diagrams). As an initial step, overlapping genes with significant modulation of expression in the pancreas, liver and lung tissues were identified in three functional annotations: cell movement (Figure 3A), cell signaling (Figure 4A), and cell death and survival (Figure 5A). In a follow-up step, the genes that had been identified by this procedure and were listed in two or more organs were further analyzed. For evaluating differences between organs, the respective ratios in gene expression were used for describing the genes’ response to the respective environment. The inclusion criterion for the selected genes was based on their significant modulation from a 1:1 ratio in gene expression.

In cell movement, 17 genes (Figure 3B) with a modulation of expression >1.5 fold were selected from 31 genes, which were listed in both pancreas, and liver (Figure 3A). Among the selected genes, six showed a ratio smaller than 0.67. Comparing lung *versus* pancreas (Figure 3C), three of the five genes were included for graphical representation. Finally, two genes (CXCL8 and STAT3) with commonality in all three organs (Figure 3D) were depicted by using the *in vitro* as well as pancreas expression levels as denominators.

In cell signaling, 25 genes were selected from 42 genes, which were shared between pancreas and liver (Figure 4A). From these, 12 genes had a modulation of expression more than 1.5 and the remaining 13 genes had less than 0.67 expression fold change (Figure 4B and 4C). When pancreas was compared with lung, TNF was the only gene identified with a significant decreased expression fold change in the lung. On the other hand, CXCL8 was also the only gene that was detected in all three organs (Figure 4D).

In cell death and survival (Figure 5A), only two genes (RRM2 and TNF) where shared between pancreas and liver. This was in stark contrast to both cell movement and cell signaling, where more genes were shared between these organs. Significantly modulated genes that were listed between pancreas and liver (1 of 2), as well as those listed between pancreas and lung (4 of 5), were represented in a single plot (Figure 5B). When comparing liver and lung, 34 genes were identified for this annotation as opposed to those occurring in similar tissues for cell signaling and cell movement. 21 of these genes had significant expression levels in the liver and lung when compared to pancreatic tissue (Figures 5C). In all three tissues, three (BIRC3, KISS1 and MYC) of four genes were profiled for their significant change in expression level (Figure 5D).

**Figure 6:**
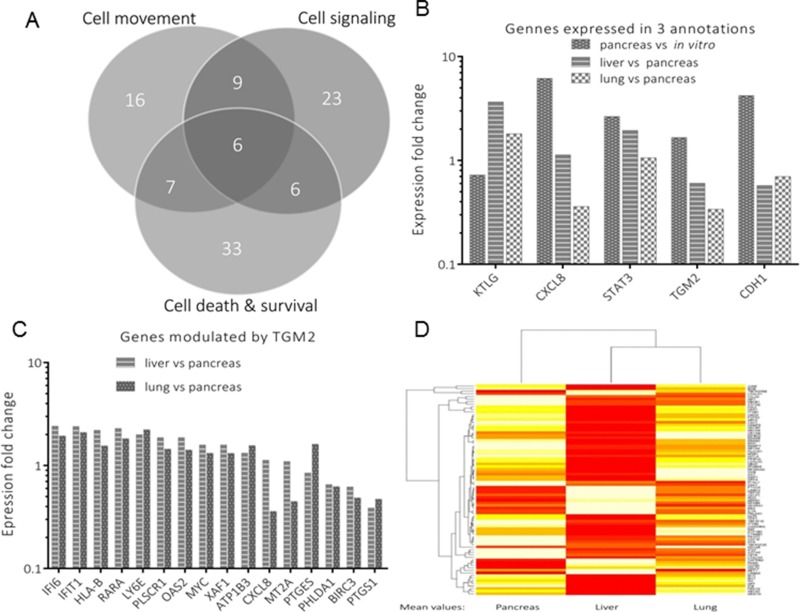
Analysis of genes attributed to three annotations **A** represents genes’ distribution (*n* = 100) for an exclusion-overlap analysis (Venn diagram), which were modulated in three functional annotations. From these, significantly modulated genes of interest are shown in **B**. Among these genes is TGM2, which modulates several downstream genes profiled in **C**. A heat map representing selected genes from the three annotations, in the three environments (pancreas, liver and lung) is shown in Figure **D**.

To identify genes of interest, those genes that were previously shared by two or more functional annotations in respective environment were further subjected to exclusion-overlap analysis, which involved three functional annotations: cell movement, cell signaling, and cell death and survival (Figure 6A). From these, a total of 22 genes were distributed in these annotations as follows; pancreas *versus* liver (*n* = 9), pancreas *versus* lung (*n* = 7) and liver *versus* lung (*n* = 6). There were six genes identified, which were listed in all three functional annotations (Figure 6B). Among these, TGM2 was one of the most significantly modulated genes and an upstream regulator of several target genes, shown in Figure 6C. Its expression level (mRNA level) in the liver was higher compared to that of lung (Figure 6B). A full list of its target genes is shown in the Supplementary Figure-1. Figure 6D represents a comparative summary in the form of a heat map for selected genes involved in the three functional annotations. This comparison was based on the genes’ mean expression levels in the three organs, highlighting their specific modulation in respect to a given environment.

**Figure 7:**
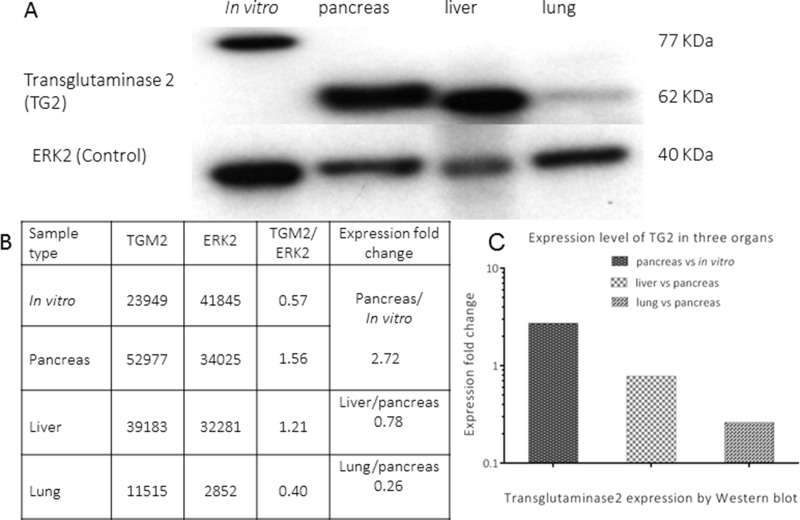
Expression of transglutaminase2 (TG2) in four environments **A** represents the expression of transglutaminase2 (TG2) and ERK2 (loading control) by Western blot, respectively, in different environments (lysate from cells growing *in vitro* and homogenates from lesions growing in pancreas, liver and lungs). The quantitative analysis of TG2 expression for each sample is shown in **B**. A comparative bar graph for TG2 modulation in pancreas, liver and lung, respectively, is shown in **C**.

At protein level, the modulation of expression of TG2 was evaluated by Western blot using samples from four environments, as indicated in Figure 7A (TG2 and ERK2). Its expression was detected at 77 and 62 KDa for *in vitro* and *in vivo* samples, respectively. Analysis of these bands showed a higher expression of TG2 in the pancreas compared to cells growing *in vitro*. When relating metastatic organs to pancreas, the expression of TG2 in liver was higher than that in lung (Figure 7C). This result was in line with the respective expression at mRNA level. A full list of the genes analyzed by Ingenuity Pathway Analysis for pancreas, liver and lung is given in Supplementary Tables 1, 2 and 3, respectively. In addition, a list of the names for genes symbols, which were analyzed by IPA is shown in Supplementary Table 4.

**Figure 8:**
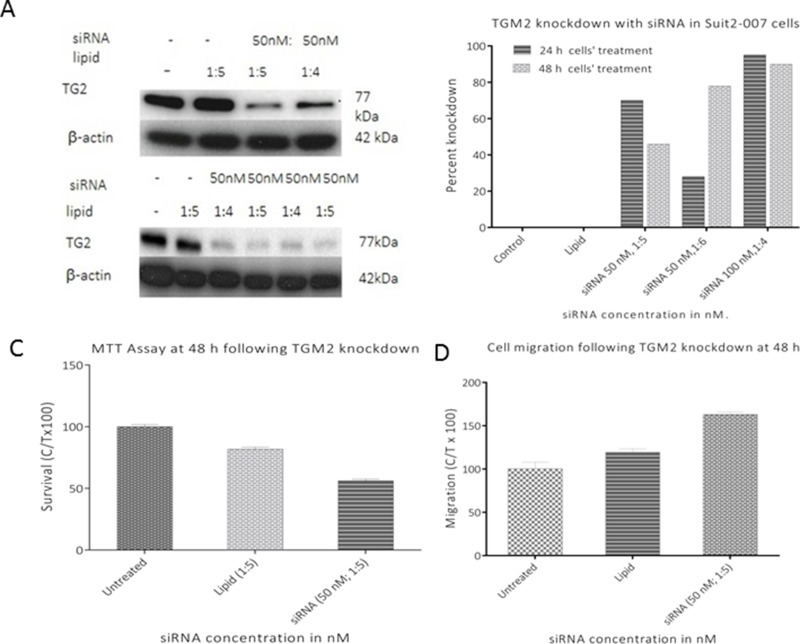
Effect of TGM2 knockdown on Suit2-007 cell proliferation and migration **A** and **B** represent western blot and RT PCR analyses of TGM2^siRNA^ induced knockdown. The effects resulting from a successful knockdown of TGM2 on cell proliferation (MTT assay) and cell migration at 48h are shown in figures **C** and **D**, respectively.

Based on the association of TGM2 with the modulation of several genes, additional experiments were performed to evaluate its role in tumor progression. TGM2 specific siRNA was designed and used for its transient knockdown. The expression of TGM2 in Suit2-007 cells following exposure for 24 and 48h time points are shown at protein (Figure 8A) and mRNA levels (Figure 8B), respectively. The full data of the RT-PCR experiment for TGM2 knockdown are shown in the Supplementary Table 5. This knockdown was further evaluated for its effect on cell proliferation and cell migration (Figures 8C and 8D), respectively. Remarkably, a transient TGM2 knockdown decreased cell proliferation by 30-40% (Figure 8C) whereas a similar treatment induced 20-40% increase in cell migration (Figure 8D).

**Figure 9:**
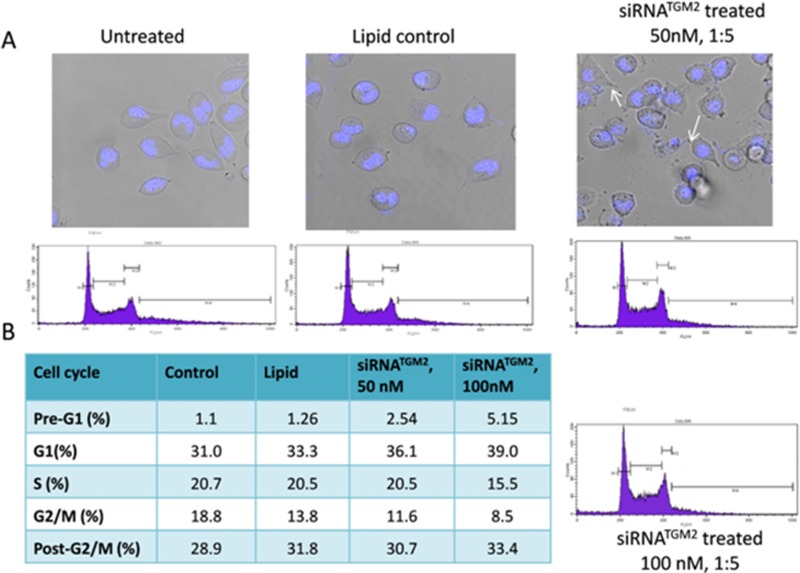
Nuclear morphology and cell cycle distribution of Suit2-007 cells following knockdown of TGM2 by siRNA **A** shows Suit2-007 cells as visualized by fluorescence microscopy for control, lipid and siRNA treated cells, respectively. Fragmented cells are visible in the Hoechst stained TGM2^siRNA^ treated cells (indicated by arrows). **B** shows results of cell cycle analysis by FACS, following TGM2^siRNA^ knockdown. The table indicates the various changes in the cell cycle due to TGM2^siRNA^ treatment with 50 and 100 nM concentrations, respectively.

Additional experiments were performed to evaluate the effect of TGM2^siRNA^ on apoptosis and cell cycle progression. For this purpose, cells were treated with TGM2^siRNA^, followed by Hoechst staining and evaluation by fluorescence microscopy (Figure 9A). TGM2^siRNA^ treated cells underwent a morphological fragmentation process, which is a characteristic of cells’ death by apoptosis. By FACS analysis, a significant dose dependent increase was observed in the Pre-G1 phase in response to 50 and 100 nM concentrations of TGM2^siRNA^ (Figure 9B). In addition, TGM2^siRNA^ treated cells exhibited a decrease in G2/M phases (9% *versus* 19%) and a slight increase in the cell fraction with post-G2/M. Further, the knockdown caused a measurable G1 blockade, which was evident from the increased G1 ratio (39%) in comparison to control (31%). This led to a decrease in the number of cells in S-phase (16% of cells), as compared to controls cells (21%; Figure 9B).

## DISCUSSION

To investigate how organ environments modulate the expression of various genes during pancreatic adenocarcinoma progression, a rat model (RNU) described previously [29] was further developed to include lung metastasis, as well as orthotopic tumor growth in the pancreas. Viral vector based plasmids used in the previous study may alter tumor cells due to genotoxicity and induce genetic changes because of their random genomic integration [30]. Our approach also differed from our earlier work, in the sense that transfection of Suit2-007 cells with luciferase reporter gene was performed with a non-viral vector plasmid that precludes genomic integration. Our current model makes it feasible to draw comparisons for different genes’ profiles in regard to organ specificity and general metastasis. Initially, there were a large number of genes showing significant modulation of expression, which resulted from the comparison between cells growing *in vitro versus* those growing *in vivo*. In fact, the change in growth from artificial surface to a natural environment was associated with >2000 genes having significant modulation of expression. This selection was refined as we were able to compare the expression levels of PDAC cells growing in liver and lung with those in pancreas. Using the expression of cells growing in pancreas as a denominator, the number of significantly modulated genes was reduced by half in the liver, and by two thirds in those growing in the lung. The number was again significantly reduced when these genes were further analyzed by Ingenuity Pathway Analysis, which yielded a series of functional annotations. From these annotations, we selected the most important ones with regard to cancer progression and thus narrowed down the number of genes of interest. The gene composition of these annotations was further reduced to 100, including only those with significant modulation in at least two organs. Finally, by exclusion-overlap analysis, only six genes showed significant modulation regarding all three functional annotations. Although each of these genes could be a promising target, we selected TGM2 for more detailed analysis. The gene TGM2 codes for tissue transglutaminase2 (TG2), a multifunctional protein that catalyzes the covalent cross-linking of proteins, in a calcium dependent manner [31]. It is one of the most significantly expressed genes in pancreatic ductal adenocarcinoma, with expression levels that correlate with tumor progression [32][31][33]. Depending on cellular localization, the cytosolic calcium as well as GTP levels, TG2 has been implicated in various cellular processes, including cell migration, differentiation, apoptosis, inflammation, and wound healing [34]. TG2 also plays a catalytic function in Ca2+ dependent transamidation of polypeptide chains involving glutamine and lysine residues. However, this function is limited when GTP binds TG2, in which case it acts as a G-protein. When TG2 is secreted to the extracellular space, it plays an adhesive role stabilizing the extracellular matrix [35]. The mechanism through which TG2 regulates cell death and survival is complex, but it is believed to involve the dual functions depending on its active (non-GTP bound) or inactive (GTP-bound) state [36]. TG2 participates in cell death, when its transamidating activity is fully operational (active form) and plays a protective role when the transamidating activity is non-operational (inactive form) [37].

In our model, TGM2 showed the highest expression in tumor cells growing in pancreatic tissues, but only 60 and 30% of these levels were detected in PDAC cells growing in liver and lung tissues, respectively. A comparison of the expression pattern of TG2 in various environments at protein level also showed an analogous modulation of expression as that occurring at mRNA level. Similar findings in TGM2 expression levels in primary and metastatic lesions have been described for colorectal cancer cell lines [38] as well as for tissues from patients with colorectal cancer [39].

In our study, transient silencing of TGM2 in Suit2-007 cells had an inhibitory effect on proliferation as well as a stimulatory effect on cell migration. The latter observation is in agreement with recent findings on TGM2 knockdown in SW480 and SW620 cells [39]. These authors described tumor cell migration/invasion in response to siRNA silencing of TGM2. We derive from these observations that reduced TGM2 levels are probably beneficial for successive colonization of metastatic target organs.

To understand the mechanism through which TGM2 inhibits cell proliferation, a transient knockdown was performed with siRNA specific for TGM2 followed by analysis of nuclear morphology and cell cycle progression. Characteristic nuclear condensation accompanied by cell DNA fragmentation was evident, supporting the involvement of TGM2 in cell death. Additionally, the silencing of TGM2 was associated with cell cycle blockade at G1/S phases of the cycle. Besides these observations, TGM2 was associated with the modulation of multiple target genes, some of which are involved in tumor progression. One of these genes is CXCL8, which was also listed among the six genes involved in three functional annotations. From literature, CXCL8 knockdown in prostate cancer cell lines (PC-3 and DU145) was associated with decreased cell proliferation and an arrest of PC-3 cells in G1 phase of cell cycle, thus preventing their entry into S-and G2/M phases [40]. This observation underscored a striking parallelism with TGM2, which influenced cell cycle progression in a similar pattern. Additionally, these two genes have been associated with epithelial mesenchymal transition (EMT), a process associated with metastasis [41][42][43].

In conclusion, we have shown that different organ environments modulate the expression of various genes during tumor progression, despite the same genetic make-up of tissues. The expression of TGM2 and similar genes of equal importance could be exploited for PDAC therapy.

## MATERIALS AND METHODS

### Cell culture

Vials containing human PDAC (Suit2-007) cells from −80°C storage were subjected to centrifugation (2500 rpm, 5 min) at room temperature in RPMI medium (enriched with 10% fetal calf serum-FCS and 1% glutamine). The mycoplasma free cells were seeded in 25 cm^2^ culture flasks and allowed to grow in standard cell culture conditions (37°C, 100% humidity, 5% CO2 in air). The cells in culture were maintained at logarithmic growth phase by passaging them twice a week.

### Transfection of Suit2-007 cells

For transfection of Suit2-007 cells, a pS/MARt DNA Vector expressing luciferase reporter gene was used. Cells’ suspension (1×10^5^) were seeded in 24 well plates in 1 ml volume and kept in standard culture conditions. The following day, a concentration of 1 μg of pS/MARt-Luciferase was diluted in 150 mM NaCl to a final volume of 50 μl. In another tube, 2 μl of jetPEI and/or GeneCelline reagent (Polyplus transfection Co.) was diluted in 150 mM NaCl to a final volume of 50 μl. The two solutions were mixed in 1:1ratio, vortexed gently and incubated at room temperature for 15 to 30 min. This mixture (100 μl jetPEI/DNA) was added drop-wise to the cells, and they were then kept in the cell culture under standard conditions. After 24h transfected cells were washed once with autoclaved PBS and their medium replaced with RPMI containing 150 μg/ml of D-Luciferin. BLI was performed in a FusionSL imager (PeqLab) using the *in vivo* luciferase expression automatic function.72h after transfection, cells were harvested and re-cultivated in culture flasks using RPMI medium containing puromycin. Puromycin selection was performed for 3 to 4 weeks with gradual increase in its concentration (0.5-1.0μg/ml). Positively transfected cells were determined by chemiluminescence test.

### Evaluation of growth potential of Suit2-007^luc^ in nude rats

Animal experiments were performed in accordance with the laws and regulations governing animal experiments in the Federal Republic of Germany, following the approval of the respective animals’ ethics committee (Regierungspräsidium Karlsruhe, Germany). Initially, eight animals (6 weeks-old) weighing 120-130 g were bought from Charles River Company (Sulzfeld, Germany). The rats were maintained in ventilated macrolon cages, under controlled conditions (22°C ± 1°C temperature, 55% humidity and 12h dark/light rhythm) in a specific pathogen free animal facility at the German Cancer Research Center (DKFZ). They were allowed unlimited access to a commercial chow and autoclaved tap water *ad libitum*. Prior to the commencement of any experiment, a seven day window was adhered to, for acclimatization. For tumor implantation, the abdominal cavity was opened under anesthesia (Isoflurane) and ∼3 × 10^6^ cells/animal were injected intraportally (via a mesocolic vein) under the microscope. Wounds were closed by suturing the musculature followed by attaching the edges of the separated skins by surgical wound clips. Tumor growth was monitored on a weekly basis by subcutaneous injection of luciferin (500μl/animal), prior to imaging by IVIS 2000 system (Perkin Elmer, US).

### Tumor implantation for gene profiling

In another set of experiments, ∼3 × 10^6^ cells/animal were injected intraportally for tumor growth in both liver and lungs. For the pancreas, ∼1.5×10^6^ cells/animal were injected orthotopically. After four weeks of tumor growth, animals were sacrificed and tumor nodules re-isolated from these organs for total RNA isolation and histological evaluation.

Total RNA was extracted as detailed in the Qiagen RNA isolation kit. Briefly, tissues weighing ∼100 mg were taken from each sample and disrupted in liquid nitrogen using a microdismembrator (Sartorius, GmbH, Germany) at 2500 vibrations/1 min. The powdered samples were homogenized with 5ml Qiazol lysis reagent and allowed to incubate at room temperature for 5 min. Genomic DNA was depleted by mixing samples with 500 μl of genomic DNAs solution. Total RNA was extracted with chloroform and washed with 70% ethanol. Total RNA was subsequently separated with RNeasy columns using RWT and RPE buffers. The concentrations of isolated RNA were determined by Nano drop spectrophotometer (NanoDrop Technologies, Wilmington, DE). In the core facility, the quality of RNA was evaluated by gel analysis using the total RNA Nano chip assay on an Agilent 2100 Bioanalyzer (Agilent Technologies GmbH, Germany).

### Probe labeling and illumina SentrixBeadChip array hybridization

Biotin-labeled cRNA samples for hybridization on Illumina Human Sentrix-12 BeadChip arrays (Illumina, Inc.) were prepared according to Illumina's recommended sample labeling procedure, based on the modified Eberwine protocol. In brief, 250 - 500 ng total RNA was used for complementary DNA (cDNA) synthesis, followed by an amplification/labeling step (*in vitro* transcription) to synthesize biotin-labeled cRNA according to the Illumina® Total Prep™ RNA Amplification Kit (Life Technologies). Biotin-16-UTP was purchased from Roche Applied Science, Penzberg, Germany. The cRNA was column purified according to the Total Prep RNA Amplification Kit, and eluted in 60-80 μl of water. The quality of cRNA was controlled using the RNA Nano Chip Assay on an Agilent 2100 Bioanalyzer and quantified by NanoDrop spectrophotometer.

Hybridization was performed at 58°C, in GEX-HCB buffer (Illumina Inc.) at a concentration of 100 ng cRNA/μl, unsealed in a wet chamber for 20h. Spike-in controls for low, medium and highly abundant RNAs were added, as well as mismatch control and biotinylation control oligonucleotides. Microarrays were washed once in High Temp Wash buffer (Illumina Inc.) at 55°C and then twice in E1BC buffer (Illumina Inc.) at room temperature for 5 min (in between washed with ethanol at room temperature). After blocking for 5 min in 4 ml of 1% (wt/vol) Blocker Casein in phosphate buffered saline Hammarsten grade (Pierce Biotechnology, Inc., Rockford, IL), array signals were developed by a 10-min incubation in 2 ml of 1 μg/ml Cy3-streptavidin (Amersham Biosciences, Buckinghamshire, UK) solution and 1% blocking solution. After a final wash in E1BC, the arrays were dried and scanned. Microarray scanning was done using an iScan array scanner. Data extraction was done for all beads individually, and outliers were excluded when > 2.5 MAD (median absolute deviation). All remaining data points were used for the calculation of the mean average signal for a given probe, and standard deviation for each probe was calculated.

### Histology

Tumor tissues (300-500 mg) from pancreas, liver and lung were harvested in 4% paraformaldehyde (PFA) at 4°C (PH 7.2). The specimens were later processed for H & E staining by embedding them in paraffin blocks. Tissue slices were rinsed twice in PBS for 15 min at room temperature and then dehydrated serially in ethanol as follows; 70% for 30 min (x2), 85%, for 60 min, 95% for 60 min, and 100% for 60 min at room temperature. Ethanol was cleared by immersing the tissues in Xylol for 60 min (x2) at room temperature. This was followed by immersion in paraffin (x3) for 60 min and formation of paraffin blocks which were stored for sectioning. Thin sections (4-5 μm) were prepared from paraffin blocks with a microtome and kept to dry overnight at 37°C for subsequent staining. The sections were then serially exposed to Xylene (x2), 100% EtOH, 95% EtOH, 70% EtOH, H_2_O (x2) respectively, for 2 min each. They were stained with Hematoxylin solution for1 min, and then rinsed with warm running tap water (15 min). Before counterstaining with eosin Y solution (1 min), sections were exposed to 95% reagent alcohol (30 sec). They were dehydrated and cleared with 95% reagent alcohol (x2), absolute reagent alcohol (x2), Xylene (x2) for 2 min in each case and mounted with resinous mounting medium.

### Western blotting

Unless otherwise indicated, reagents for the following experiments were purchased from Serva Electrophoresis, GmbH and Santa Cruz Biotech, USA (antibodies) and In vitrogen (siRNA and primers). A cell density of 1.5× 10^5^/2ml medium/well was seeded in 6 well plates and kept in standard culture conditions for 24h. The cells were then exposed to 50 nM siRNA^TGM2^ in X-tremeGENE^TM^ reagent. Lysates and tumor homogenates from *in vitro* and *in vivo* samples, respectively were prepared and their protein concentrations determined by Roti Nanoquant. Protein aliquots were mixed proportionately with a loading dye (NuPage) and 2 μl of 1M of dithiothreitol (DTT). The samples were denatured on a heat block for 5 min (at 99°C) with gentle vortexing and loaded (15 μg/ml) onto the wells of precast gels, 4-20% polyacrylamide, in 1x Laemmli buffer and separated by electrophoresis for 60 min at 150-200 V.

Transfer was performed following the activation of PVDF western blotting membrane (Roche diagnostics GmbH) using methanol (1 sec) followed by washing in double distilled water (1 min) and immersion into the transfer buffer. The gel and membrane were sandwiched between transfer pads and then stacked intact in the electrophoresis device, which was run at 35V for 1h.

Membranes were blocked with blocking solution (5% skimmed milk in 1x TBST) for 1h with constant agitation, a process that was followed by overnight incubation at 4°C with primary antibodies, β-actin (1:3000) and transglutaminase2 (1:5000). The following day the membranes were washed (x3) for 15 min with a washing buffer (1x TBS containing 0.1% v/v Tween 20) and incubated at room temperature for 1h with secondary antibody (1:10000). After washing (x3), membranes were exposed to western blot chemiluminescence reagents, sealed with transparent polythene paper and exposed to an X-ray film. The film was developed in Optimax X-ray film processor (PROTEC GmbH & Co, Germany) after exposure for 30 sec, 1 min and a 10 min interval for quality bands. The films were then scanned and analyzed by Image J software.

### Real-time PCR for TGM2 expression

A cell suspension containing 1-1.5×10^5^ cells/well was seeded in 6-well plates and at 24h; they were treated with a TGM2 specific siRNA. After 24h and 48h, cells were harvested and total RNA was extracted with the Qiagen RNAs extraction kit. The concentrations of the RNA in treated and control samples were quantified with NanoDrop^TM^ spectrophotometer (ThermoFischer Scientific, Germany). RNA of good quality (260/280 ratio in the range of 1.9-2) was achieved and subsequently used in the synthesis of complementary DNA (cDNA). A reaction mixture (total volume of 20μl) comprising RT buffer (1x), RNAse inhibitor (10 units), 1μl dNTPs (10mM), 1μl oligo-dT-primer (10μM) and Maxima reverse transcriptase (200 units) enzyme (ThermoScientific, Damstadt) was prepared in PCR tubes. Complementary DNA was synthesized in a reaction with defined conditions (at 50°C for 30min, 85°C for 5min and 10°C) in the PCR machine (PTC-200, Peltier Thermal Cycler) for 60min.

After cDNA synthesis, the expression level of the two genes was evaluated using the LightCycler 480 Real-Time PCR system (Roche Life Science, Germany). Aliquots (2μl) of cDNA prepared from RNAs (25-100ng) per treated sample were added in triplicate wells (in 384-well-plates) to a total volume of 10μl/sample and amplified. The respective expression level of GAPDH was used as a reference gene to normalize the data. Corresponding changes in the gene expression levels of TGM2 were calculated by the 2-∆∆CT method.

### MTT assay

An optimized cell density (1.5× 10^4^/2ml medium/well) was seeded in 6 well plates and kept in standard culture conditions for 24h. The cells were then exposed to 50 and 100 nM siRNA^TGM2^ in X-tremeGENE^TM^ reagent for 48h. After this period of incubation (37°C), 200μl/well of MTT solution was added to well plates. The plates were incubated for another 3h in the cell culture incubator, after which 2ml/well of 2-propanol solution containing HCL (0.04N) was added. Absorbance was measured by ELISA reader (Biotech instruments¸ Germany) at 540 nm (excitation) and 690 nm (emission) wavelengths.

### Cell migration

In a cell migration assay, about 1.5× 10^5^ cells were seeded in 6 well plates and kept under standard conditions for 24h. The cells were then treated with siRNA^TGM2^ 50nM and plates incubated in the cell culture incubator for 5 h after which, cells were trypsinized and counted as mentioned before. A cell suspension of 8.0 × 10^4^ − 10 × 10^5^ cells in 300μl/ml Opti-MEM^M^ medium was added to each cell inserts (ThinCerts^TM^, Greiner Bio-one) for the treated sample and controls. The inserts were then suspended in a 24 well plate containing 700 μl complete RPMI medium. The plates were incubated for 48h after which 140 μl of cell titer blue was added and further incubated for 3 - 4h. The inserts were discarded and the number of cells that migrated into the lower compartment was determined by Elisa reader at 560 excitation and 585 nm emission wavelengths.

### Cell cycle assay

For evaluating the effects of TGM2 knockdown with siRNA, a cell cycle experiment was performed. Suit2-007 cells were seeded (2.5-3.5×10^5^ cells/well) in a 6 well plate and after 24h of attachment, cells were treated with siRNA^TGM2^ (50, and 100 nM concentrations), and kept in the standard cell culture incubator for 48h. Later, the cells were harvested and 2×10^5^ cells were re-suspended in 100μl of PBS followed by addition of 70% ice cold ethanol for fixation.

The cell suspension was then incubated for 2h at 4°C and washed of ethanol by centrifugation using PBS. The cell pellets were re-suspended in 300μl PBS containing RNaseA (1mg/ml) to get rid of their RNA content and incubated at 3°C for 30min. After incubation, PI (50 μg/ml) was added and cells were allowed to incubate at room temperature for 15-30min. Analysis was performed with a FACS Calibur (BD Biosciences, Germany) at 10-15 thousand events to determine the distributions of cells in different phases of cell cycle (G0/G1, S and G2/M).

### Hoechst staining

The mode of cell death occurring following siRNA^TGM2^ treatment, was analyzed by fluorescence microscopy by Hoechst stain. About 2.5× 10^5^−3×10^5^/cells were seeded (200μl/well) in four wells of the 8 chamber glass boxes (Nunc™ Lab-Tek® Chamber Slide™, ThermoFischer Scientific) and treated with a solution of siRNA^TGM2^ (50nM) in X-tremeGENE^TM^ transfection reagent. After 24h of incubation in the standard culture cell incubator, the medium was exchanged with a fresh medium containing 2 drops (40-50μl) per ml of Hoechst dye. The glass chambers were then incubated at room temperature for 20 min after which imaging was performed with Leica SP5 fluorescence microscope (Leica Biosystems GmbH, Germany). The captured images were processed by Image J software. For each of these assays, three independent experiments were performed to confirm the findings.

### Statistical analysis

Data from the chip array experiment were pre-processed, normalized and then analyzed with Chipster (empirical Bayes) (±1.5 cut-off fold expression change and *p* < 0.05). Significant expression fold change for individual groups were derived from the respective ratios using vitro and pancreas mean expression as denominators for primary as well as secondary organs (liver and lung), respectively. The second phase of analysis was performed with IPA platform to generate series of functional annotations. Functional annotations for respective organs were selected based on significant p values and Z-scores (±2) and analyzed by exclusion-overlap analysis (Venn diagrams) (*http://bioinformatics.psb.ugent.be/webtools/Venn/*). Final plots were done using Adobe illustrator and Graph Pad prism software.

## SUPPLEMENTARY MATERIAL FIGURE AND TABLES



## References

[1] Jemal A, Siegel R, Xu J WE (2010). Cancer statistics, 2010. CA Cancer J Clin.

[2] Bosetti C, Bertuccio P, Malvezzi M, Levi F, Chatenoud L, Negri E LVC (2013). Cancer mortality in Europe, 2005-2009, and an overview of trends since 1980. Ann Oncol.

[3] Malvezzi M, Bertuccio P, Levi F, La Vecchia C NE (2014). European cancer mortality predictions for the year 2014. Ann Oncol.

[4] Malvezzi M BP, Rosso T, Rota M, Levi F, La Vecchia C NE (2015). European cancer mortality predictions for the year 2015: does lung cancer have the highest death rate in EU women?. Ann Oncol.

[5] Stark A, Eibl G (2015). pancreatic ductal adenocarcinoma.

[6] Guan X (2015). Cancer metastases: Challenges and opportunities. Acta Pharm Sin B.

[7] Yoshida BA, Sokoloff MM, Welch DR R-SC (2000). Metastasis-suppressor genes: a review and perspective on an emerging field. J Natl Cancer Inst.

[8] Kamisawa T, Isawa T, Koike M, Tsuruta K OA (1995). Hematogenous metastases of pancreatic ductal carcinoma. Pancreas.

[9] Disibio G FS (2008). Metastatic patterns of cancers: results from a large autopsy study. Med, Arch Pathol Lab.

[10] Steeg PS (2016). Targeting metastasis. Nat Rev Cancer [Internet].

[11] Steeg PS (2006). Tumor metastasis: Mechanistic insights and clinical challenges. Nat Med.

[12] Weaver VM, Petersen OW, Wang F, Larabell CA, Briand P, Damsky C, Bissell MJ (1997). Reversion of the Malignant Phenotype of Human Breast Cells in Three-Dimensional Culture and In Vivo by Integrin Blocking Antibodies. J Cell Biol [Internet].

[13] Nelson CM, Bissell MJ, Division LS, Berkeley L, Stricker J, Sabass B, Schwarz US, Gardel ML (2010). NIH Public Access. J Phys (main title).

[14] Provenzano PP, Hingorani SR (2013). Hyaluronan, fluid pressure, and stromal resistance in pancreas cancer. Br J Cancer [Internet].

[15] Bamford S, Dawson E, Forbes S, Clements J, Pettett R, Dogan A, Flanagan A, Teague J, Futreal PA, Stratton MR, Wooster R (2004). The COSMIC (Catalogue of Somatic Mutations in Cancer) database and website. Br J Cancer [Internet].

[16] Xie D, Xie K (2015). Pancreatic cancer stromal biology and therapy. Genes Dis [Internet].

[17] Nielsen MFB, Mortensen MB, Detlefsen S (2016). Key players in pancreatic cancer-stroma interaction: Cancer-associated fibroblasts, endothelial and inflammatory cells. World J Gastroenterol.

[18] Ungefroren H, Sebens S, Seidl D, Lehnert H, Hass R (2011). Interaction of tumor cells with the microenvironment. Cell Commun Signal [Internet].

[19] Lunardi S, Muschel RJ BT (2014). The stromal compartments in pancreatic cancer: Are there any therapeutic targets?. Cancer Lett.

[20] Shen W, Tao G, Zhang Y, Cai B, Sun J, Tian Z (2017). TGF-β in pancreatic cancer initiation and progression: two sides of the same coin. Cell Biosci [Internet].

[21] De Wever O, Mareel M (2003). Role of tissue stroma in cancer cell invasion. J Pathol [Internet].

[22] Lee J, Condello S, Yakubov B, Emerson R, Caperell-Grant A, Hitomi K, Xie J, Matei D (2015). Tissue transglutaminase mediated tumor-stroma interaction promotes pancreatic cancer progression. Clin Cancer Res.

[23] Evan GI, Hah N, Littlewood TD, Sodir NM, Campos T, Downes M, Evans RM (2017). Re-engineering the Pancreas Tumor Microenvironment: A &quot;Regenerative Program&quot; Hacked. Clin Cancer Res [Internet].

[24] Quail D. F., Joyce JA (2013). Microenvironmental regulation of tumor progression and metastasis. Nat Med.

[25] Shimizu K (2008). Pancreatic stellate cells: Molecular mechanism of pancreatic fibrosis. J Gastroenterol.

[26] B JM (1991). Molecular themes in oncogenesis. Cell.

[27] Raimondi S, Lowenfels AB, Morselli-Labate AM M-, neuve P PR (2010). Pancreatic cancer in chronic pancreatitis; aetiology, incidence, and early detection. Best Pr Res Clin Gastroenterol.

[28] Hidalgo M, Cascinu S, Kleeff J, Labianca R, Löhr JM, Neoptolemos J, Real FX, Van Laethem JL, H V (2015). Addressing the challenges of pancreatic cancer: future directions for improving outcomes. pancreatology.

[29] Eyol E, Murtaga A, Zhivkova-Galunska M, Georges R, Zepp M, Djandji D, Kleeff Jö, Berger MR, Adwan H (2012). Few genes are associated with the capability of pancreatic ductal adenocarcinoma cells to grow in the liver of nude rats. Oncol Rep.

[30] Pannell D, Ellis J (2001). Silencing of gene expression: implications for design of retrovirus vectors. Rev Med Virol.

[31] Mehta K, Han A (2011). Tissue Transglutaminase (TG2)-induced inflammation in initiation, progression, and pathogenesis of pancreatic cancer. Cancers (Basel).

[32] Ellenrieder V, König A, Seufferlein T (2016). Current Standard and Future Perspectives in First- and Second-Line Treatment of Metastatic Pancreatic Adenocarcinoma. Digestion.

[33] Iacobuzio-Donahue CA, Ashfaq R, Maitra A, Adsay NV, Shen-Ong GL, Berg K, Hollingsworth MA, Cameron JL, Yeo CJ, Kern SE, Goggins M, Hruban RH (2003). Highly Expressed Genes in Pancreatic Ductal Adenocarcinomas: A Comprehensive Characterization and Comparison of the Transcription Profiles Obtained from Three Major Technologies. Cancer Res.

[34] Nurminskaya MV BA (2012). Cellular functions of tissue transglutaminase. Int Rev Cell Mol Biol.

[35] Verderio EA, Johnson T GM (2004). Tissue transglutaminase in normal and abnormal wound healing: review article. Amin Acids.

[36] Fésüs L SZ (2005). Transglutaminase 2 in the balance of cell death and survival. FEBS J.

[37] Milakovic T, Tucholski J, McCoy E, Johnson GVW (2004). Intracellular Localization and Activity State of Tissue Transglutaminase Differentially Impacts Cell Death. J Biol Chem.

[38] Zirvi KA, Keogh JP, Slomiany A SB (1991). Transglutaminase activity in human colorectal carcinomas of differing metastatic potential. Cancer Lett.

[39] Cellura D, Pickard K, Quaratino S, Parker H, Strefford JC, Thomas GJ, Mitter R (2016). Europe PMC Funders Group MiR-19-mediated inhibition of Transglutaminase-2 leads to enhanced invasion and metastasis in colorectal cancer.

[40] Singh RK, Lokeshwar BL (2009). Depletion of intrinsic expression of Interleukin-8 in prostate cancer cells causes cell cycle arrest, spontaneous apoptosis and increases the efficacy of chemotherapeutic drugs. Mol Cancer.

[41] Zhou N, Lu F, Liu C, Xu K, Huang J, Yu D, Bi L (2016). IL-8 induces the epithelial-mesenchymal transition of renal cell carcinoma cells through the activation of AKT signaling. Oncol Lett.

[42] Fernando RI, Castillo MD, Litzinger M, Hamilton DH, Palena C (2011). IL-8 signaling plays a critical role in the epithelial-mesenchymal transition of human carcinoma cells. Cancer Res.

[43] Ayinde O, Wang Z, Griffin M, Ayinde O, Wang Z, Griffin M, Ayinde O, Wang Z, Griffin M (2017). Tissue transglutaminase induces Epithelial-Mesenchymal-Transition and the acquisition of stem cell like characteristics in colorectal cancer cells. Oncotarget [Internet].

