# Supplementary material for: The expression of genes contributing to pancreatic adenocarcinoma progression is influenced by the respective environment
Source: Genes Cancer. 2018 Mar;9(3-4):114–29. doi: 10.18632/genesandcancer.173 (PMC6086001; doi:10.18632/genesandcancer.173)
Supplement: Supplementary file 1 [file ganc-09-114-s001.pdf]

The expression of genes contributing to pancreatic adenocarcinoma progression is influenced by the respective environment – Sagini et al

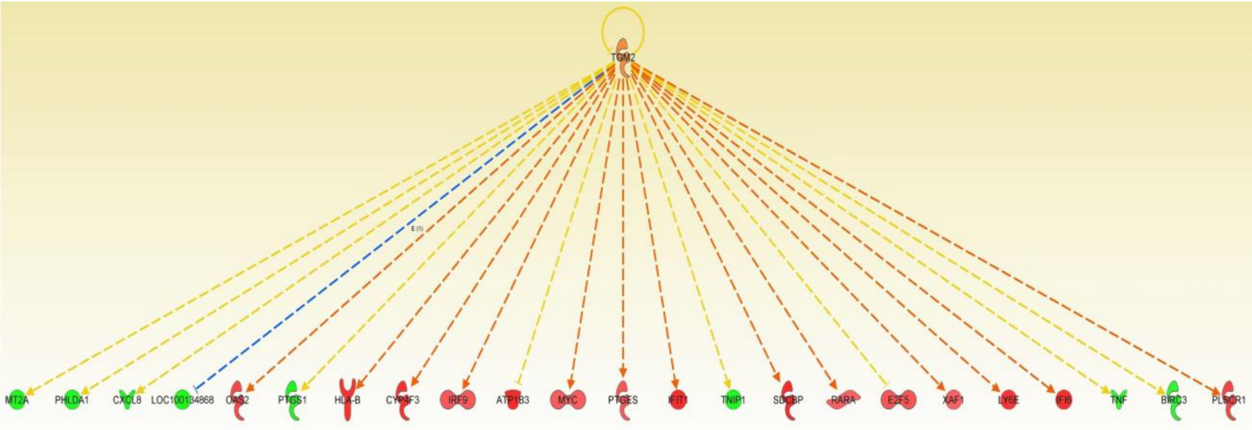

Supplementary Figure 1: Target genes regulated by TGM2. Figure represents 24 genes regulated by TGM2, which were obtained from Ingenuity Pathway Analysis. As indicated, 9 genes (marked red) are down-regulated by TGM2. On the contrary, 15 genes (marked red) are up-regulated by TGM2.

Supplementary Table 1: Functional annotations of genes from Suit2-007 cells growing in pancreatic environment

Categories[a]	Diseases or Functions Annotation[b]	p-Value[c]	Predicted activation[d]	Activation Z-score[e]	Number of genes[f]
Cell movement	Cell movement	1,56E-11	increased	2,199	LAMB3, CEACAM6, CCL20, AGR2, MUC1, CXCL1, LAMA3, LCN2, COL17A1, CXCL8, AIF1, MMP7, CEMIP, JUP, SOD2, S100A4, PDGFA, NDRG1, SGK1, IGFBP3, DDR1, IL1A, CDKN1A, NREP, SEMA3E SERPINA3, SDC4, ALPP, CX3CL1, NFKBIA, ANXA3, CDH1, CDCP1, CRYAB, TUBB2B, FOXQ1, SLPI, F3, GRINA, ITGA2, ARPIN/C15orf38-AP3S2, SPTLC1, IL10, TSC22D3, LAMC2, TCAF1, CDH3, MX1, LEP, ZC3H12A, PMP22, IL32, FAM83H, EFNA1, PATJ, CEBPB, SERPINA5, PTK6, EPHB6, JUND, TNFSF14, ERBB3, TNFRSF25, FCAR, CXCL16, HLA-A, CEACAM1, FAT1, AHR, CSF2RA, CLDN7, MAPK13, FERMT1, TCAF2, MST1R, CD99, PTP4A2, PHLDA1, DEFB1, RHOB, TNFSF15, CD44, CSF2, SERPINB5, TGM2, SRC, ITGA6, TNC, HNRNPA2B1, RHOD, SKI, KISS1, TACSTD2, GNAI2, CXCL2, NFKB2, TAGLN2, TNF, CD74, PTPRK, STAT3, ARHGAP21, VEGFA, MYH9, SAA1, F11R, PDCD4, IQGAP1, DCN, MAPK8IP3, STC1, ADAM15, LTBP2, HOOK1, CST3, EPHA1, TIMP2, LPAR2, CORO1A, CLDN3, MYO1C, ANGPTL4, SPHK2, NME1, COL18A1, PRMT1, FAF1, EZH2, SPRY2, LASP1, KITLG, CDK4, DPAGT1, LPAR1, F2R, NRP1, SELP, PBK, HNRNPK, JUN, HMMR, TUBA1C, ST3GAL5, RUVBL2, ACP1, KLK3, CDKN3, CITED2, SKP2, IGF2BP3, KIAA1524, CAV1, NET1, CDX2, CDK1, ID2, CYR61, SDCBP, HSPD1, TGFBI, PPARG, ANXA2, TMPO, VIPR1, NR5A2, EGR1, RGS4, FOSB, DNAJB6, ADRB2, PTTG1, TPM3, MB, CTGF, KPNA2, TNFRSF1B, FOS, THBS (178/326)
Cell-to-Cell Signaling and Interaction	Interaction of tumor cell lines	2,40E-10	increased	2,559	MUC1, LAMA3, CXCL8, MUC16, IGFBP3, DDR1, CX3CL1, NFKBIA, CDH1, CDCP1, F3, ITGA2, LAMC2, CDH3, LEP, SERPINA5, EPHB6, ARID1A, ERBB3, TNFRSF25, HLA-A, CD99, PHLDA1, CD44, SERPINB5, TGM2, SRC, ITGA6, TNC, CD68, CXCL2, TNF, DYSF, STAT3, ARHGAP21, VEGFA, F11R, HYAL1, DCN, ADAM15, SPTAN1, NME1, PRMT1, KITLG, INHBB, F2R, SELP, JUN, HMMR, ACP1, KLK3, ERP29, CAV1, NET1, PPARG, ANXA2, VIPR1, MAD2L1, FOS, THBS1(60/109).
Gene expression	Expression of RNA	6,19E-09	increased	2,572	AGR2, SOD2, PDGFA, IL1A, CDKN1A, IRF2BPL, BAMBI, KMT2E, APOBEC3G, KLK6, HOXA5, NFKBIA, FOXJ1, CDH1, CRCP, BLZF1, CREB1, NFIB, TRIM13, IL10, TSC22D3, FOXO4, AIRE, CDH3, ZC3H12A BHLHE40, CEBPB, ONECUT2, ARID1A, JUND, TNFSF14, ZNF148, TNFRSF25, IRF9, JAG1, CAPRIN2, AHR, MAGED1, TSC22D1, MNT, MYBL1, BTG1, LRRFIP1, TOB1, STAT2, FARSB, HNF1B, RHOB, GABPB2, TNFSF15, CD44, LILRB1, CSF2, TGM2, SRC, TNIP1, ITGA6, HNRNPA2B1, RBM14, DNTTIP1, SKI, CFLAR, DDIT3, FLII, LDOC1, NFAT5, NFKB2, TNF, PTPRK, STAT3, NFIX, EIF4A2, GSN, VEGFA, MAFF, PDCD4, IQGAP1, ADIRF, DCN, TNFAIP3, MED15, KLF9, LPAR2, UBR5, CBX2, TAF9, NME1, FAF1, EZH2, SPRY2, MELK, ATAD2, NKX3-1, ZFP36, INHBB, LPAR1, F2R, SFR1, HNRNPK, JUN, RUVBL2, ARF4, PHGDH, RBM4, RXRA, UHRF1,TIPARP, PLK1,

					ERP29, SUV39H1, CITED2, ARID5B, MED27, PAGR1, IGF2BP3, GMNN, BIRC5, CAV1, GTF2A2, PSRC1, TXNRD1, EIF5, CDK2, TRIP13, CDX2, ID2, CYR61, RAE1, PPARG, DHX9, NR5A2, STC2, CCND3, EGR1, FOXL2, FZD2, FOSB, PTTG1, RPL6, HNRNPA1, ALYREF, TNFRSF1B, FOS (143/341)
Cellular assembly & organization, DNA replication, recombination &repair	Formation of nuclear foci	3,33E-05	increased	3,142	CCNB1, NKX3-1, RUVBL2, RFWD3, CDK1, CCND3(6/24)
Cell death &survival	Cell death of squamous cell carcinoma cell lines	4,18E-05	increased	2,414	JUP, CD44, KISS1, CFLAR, DDIT3, TNF, STAT3, PDCD4, PLK1, BIRC5, RRM2, FBXO5 (12/24)
Cell death & Survival	Cell viability of ovarian cancer cell lines	1,69E-04	increased	2,215	SOD2, SLPI, JAG1, HNF1B, SRC, CLDN3, JUN (7/17)
Cancer, organismal injury & abnormalities	Growth of tumor	2,18E-07	decreased	-2,198	MUC1, IGFBP3, CDKN1A, BAMBI, ZMAT3, IL10, LEP, BIK, CEACAM1, JAG1, CSF2RA, SSTR2, MST1R, RHOB, CD44, CSF2, SERPINB5, SRC, SKI, DDIT3, TNF, STAT3, VEGFA, PTPRA, DCN, ADAM15, LPAR2, PPP2CA, KITLG, NKX3-1, INHBB, CDK4, KLK3, RXRA, PLK1, PEG10, SKP2, BIRC5, CAV1, ID2, CYR61, EGR1, SLC3A2. FOS, THBS1 (45/59)
Infectious diseases	Viral infection	1,44E-12	decreased	-2,198	HBA1/HBA2, LCN2, CXCL8, CLIP3, S100A4, SLC2A3, SGK1, ANO1, IL1A, IL23A, ALPP, APOBEC3G, NFKBIA, BIRC3, HMGCS1, MAGT1, TUBA3C/TUBA3D, SLPI, HIST1H2BD, CREB1, HNRNPU, CRYAB, F3, PDE4C, ARL16, IL10, MX1, TMEM132A, LEP, ZC3H12A, IL32, ARHGAP32, NUBPL, SERPINA5, PLOD2, EPHB6, ARID1A, , HIST2H2AA3/HIST2H2AA4, ZNF148, IRF9, HLA-A, CSF2RA, AP2B1, MST1R, TNFRSF14, WNT10A, CLK1, CYP3A5, PSMD12, STAT2, DEFB1, RHOB, GABPB2, F5, SHCBP1, AP1G2, CD44, CSF2, BAG6, OPTN, KANSL1, PDE4A, HSPA6, FUT2, TCIRG1, HLA-B, FLII, TAGLN2, TNF, PSENEN, CD74, DYSF, STAT3, ARHGAP21, F11R, SRRM2, DUSP3, PPM1K, PTGS1, LDLR, COPG1, MED15, STXBP2, CST3, HPGD, SPTAN1, TIMP2, TMEM63A, LPAR2, CLDN3, NUDCD3, UBR5, DCLK1, PDXK, POLR1E, SNRPF, CDC42EP3, ERCC1, ADK, PDSS1, MND1, HSPA4, EZH2, SPRY2, SF3B6, SRRT, VNN2, SAFB, CDK4, F2RNDUFAF2, PBK, DIMT1, TKT NUDT11, HNRNPK, JUN, , TUBA1C, ST3GAL5, RUVBL2, G3BP2, EXOSC3, NCKAP1, MT2A, RXRA, PLK1, PSMA3, TUBG1, UBE2L6, MED27, SNU13, CAV1, PSMA1, UNG, CDK2, RRM2, KRT18, HSPD1, PPARG, RANBP1, DHX9, MGLL, ANXA2, ARHGAP23, TYMS, CAV2, AP1S1, TUBB, TUBA1A, EGR1, UBE2E1, DUSP1, ADRB2, PTTG1, GINS4, PSMC4, TNFRSF1B (156/362)
Cellular	Alignment of	6,68E-06	decreased	-2,976	NCAPG2, SMC4, KIFC1, KIF20A, BIRC5, KIF2C, DLGAP5, SGO1 (8/12)

assembly & organization, DNA replication, recombination, and repair	chromosomes				
Cell cycle	M phase of tumor cell lines	6,89E-05	decreased	-2,240	SVIL, GNAI2, STX16, CDT1, PLK1, BIRC5, KIF20A, KIFC1, TRIP13, FBXO5, MAD2L1, PTTG1, MCM7, SPDL1(14/29)
Cell cycle	Cell cycle progression of cervical cancer cells	6,93E-05	decreased	-2,138	HYPK, AHR, PLK1, BIRC5, BANF1, NET1, CDK1, MAD2L1, TCP1 (9/29)
DNA replication, recombination, and repair	Double-stranded DNA break repair	9,29E-05	decreased	-2,135	FAM175A, TDP1, ARID1A, TNKS1BP1, ERCC1, EZH2, G6PD, EXO1, SUV39H1, CDK1, KPNA2(11/30)
Cell cycle	Interphase of bone cancer cells	9,50E-05	decreased	-2,343	NDRG1, CDH1, LEP, JAG1, MST1R, CD44, TGM2, TNF, STAT3, ARHGAP21, VEGFA, HOOK1, PRMT1,HNRNPAB, NRP1, CAV1, PTTG1, CTGF(18/20)
Cellular development	Epithelial-mesenchymal transition of tumor cells	1,05E-04	decreased	-2,678	CDKN1A, BHLHE40, NEK11, ERCC1, PLK1, CDK2 (6/10)
Cell cycle	Re-entry into interphase	1,09E-04	decreased	-2,121	KIFC1, NUSAP1, KIF20A, BIRC5, PLK1, NME1, CCNB1, STX16, MYH9, GNAI2, SVIL, RHOB, INF2, CDKN1A (15/36)

[a] Categories, are broader functions under which various gene cluster a were annotated by Ingenuity pathway analysis.

[b] Diseases or functions annotations represent predicted biological functions of gene clusters derived Ingenuity Pathway Analysis.

[c] p-Value is the calculated probability derived from individual gene cluster in a defined annotation for the respective gene group.

[d] Predicted activation is the overall activation state (increased or decreased) of the functional annotation for respective genes in a given gene cluster.

[e] Activation Z-score is a defined quantity, which determines whether a biological function has significantly more “increased” predictions than “decreased” predictions (Z>0) or vice versa (Z<0).

[f] Number of genes represent selected genes based on significant modulation of expression versus the total number of genes for respective functional annotation.

Supplementary Table 2: Functional annotations of genes from Suit2-007 cells growing in liver environment

Categories[a]	Diseases or Functions Annotation[b]	p-Value[c]	Predicted activation[c]	Activation Z-score[e]	Number ofgenes[f]
Cell-to-cell signaling &interaction	Interaction of tumor cells	1,23E-13	increased	2,191	LGALS4, KLK5,RHOU,DCN, LGALS2,APP, NR1H4, KITLG, CXCR4, CAV1, ERBB3, TNFRSF25, ITGB1, ANXA2,CDH1, HYAL1, ITGB2, ALCAM, SDC1, ARID1A, LAMA3, PKM, PHLDA1, TNFRSF6B, IRS1CDCP1, EPHB6, CLDN2, LAMC2 (28/77)
Cellular movement, immune cell trafficking	Leukocyte migration	3,58E-05	increased	3,039	DEFB1, GDF15, CD74, S100A9, PPBP, CRP, SERPINA1, APP, KITLG, CXCR4, CXCL6, EDNRB, AIF1, CCL20, SAA1, ITGB1, ITGB2, ALCAM, ENG, ADORA1, LGALS1, TNFRSF6B, IL1A(23/49)
Inflammatory response	Inflammatory response	5,19E-06	increased	2,423	DEFB1, CD74, S100A9, PPBP, CRP, SERPINA1, APP, KITLG, CXCR4, CXCL6, SERPING1, EDNRB, AIF1, CCL20, SAA1, HYAL1, MGLL, ITGB2, TNFAIP6, ENG, ADORA1, LGALS1, TNFRSF6B, IL1A, ADAM8(25/49)
Cell-to-cell signaling and interaction	Activation of cells	3,91E-05	increased	2,011	LGALS4, HLA-DPA1, CRP, APP, B2M, CXCR4, CXCL6, SERPING1, ATP2A2, CDH1, TNFSF10, ITGB2, PKM, TNFRSF6B, IL23A, IL1A, LTB (17 /53)
Cellular movement	Homing of cells	1,84E-04	increased	2,998	DEFB1, CD74, RHOU, S100A9, PPBP, SERPINA1, APP, KITLG, CXCR4, CXCL6, MDK, EDNRB, AIF1, CCL20, CAV1, SAA1, ITGB1, ANXA2, ITGB2, ADORA1, LGALS1, TNFRSF6B, FOSL1 (23/48)
Cellular growth and proliferation	Colony formation of breast cancer cell lines	5,90E-04	increased	2,044	FGFR3, SERPINA1, ELF3, IL1A (4/15)
Neurological, skeletal &muscular disorders	Neuromuscular disease	5,54E-05	increased	2,449	FGG, HLA-DRA, GABRP, HLA-DRB1, CD74, FGFR3, ALDH6A1, MT1H, HLA-DQA1, HLA-B, CRP, SERPINA1, IFIT1, APP, USP2, HLA-DMA, SERPING1, ALOX5, ETFB, ATP2A2, DPAGT1, GLRB, MX1, PLSCR4, FOXN3 DNAJB6, ISG15, ANXA2, NDRG2, HNRNPDL, TUBB2B, ALCAM, LY6E, ADRA1B, PKM, CSRP2, LAMB1,ADORA1, ARRB1, MAOB, AP1S2, PTGS1, IL1A, COL13A1 (48/106)
Neurological disease	Progressive motor neuropathy	3,02E-07	increased	2,449	HLA-DRA, TSPAN8, NNMT, GABRP, HLA-DRB1, CD74, FGFR3, HLA-DQA1, HLA-B, IFIT1, APP, PDGFC, FUCA1, HLA-DMA, SERPING1, ALOX5, MX1, DNAJB6, ISG15, ANXA2, GADD45A, NDRG2, HNRNPDL, SHROOM3, TUBB2B, ALCAM, LY6E, ADRA1B, ADORA1, ADIRF, ARRB1, MAOB, PFKP, AP1S2, PTGS1, IL1A (36/81)

Cell death and survival	Cell death	3,68E-16	decreased	-2,162	GSTA2, LGALS4, GSTA1, DEFB1, IFI27, GABRP, HLA-DRB1, GDF15, CD74, FGFR3, S100A9, LMO4, PPBP,HLA-DRB4, LCN2, SEMA6A, LGALS2, CRP, APP, NR1H4, USP2, KITLG, B2M, PI3, IFI6, PDGFC, CXCR4, MMP7, IGFBP7, MDK, ALOX5, MT1F, ARF4, ATP2A2, ITM2B, ID2, EDNRB, NAMPT, SCIN, IFIT2, NDRG1, PPM1A,HBA1/HBA2, CHMP5, WSB1, PLSCR1, MX1, TCP1, KLK6, CAV1, ANTXR1, AKR1C3, NFKBIZ, SAA1, ERBB3, TNFRSF25, ITGB1, ISG15, ADGRL2, ANXA2, ANXA4, CDH1, GADD45A, WDR19, HYAL1, NDRG2, TNFSF10, ADM, PDZK1, ITGB2, NEK11, MYC, ALCAM, GPR37, ZFAND6, DUSP1, SDC1, KRT18, ENG, THOC2, HIPK2, PKM, PHLDA1, ADORA1, LGALS1, TNFRSF6B, SULF2, PHLDA2, CAPRIN2, LMNA, ARRB1, IRS1, FOSL1, MAOB, MLLT11, TRPV2, CDC25B, CDCP1, PMP22, EPHB6, LOC102724788/PRODH, PTGS1, IL1A (103/306)

[a]Categories are broader functions under which various gene clusterare annotated by Ingenuity pathway analysis;

[b]Categories orFunctions annotations represent predicted biological functions of gene clusters derived Ingenuity Pathway Analysis

[c]p-Value is the calculated probability derived from individual gene cluster in a defined annotation for the respective gene group.

[d]Predicted activation is the overall activation state (increased or decreased) of the functional annotation for respective genes in a given gene cluster

[e]Activation Z-score ; is a defined quantity, which determines whether a biological function has significantly more “increased” predictions than “decreased” predictions (Z>0) or vice versa (Z<0).

[f]Number of genes, represent selected genes based on significant modulation of expression versus the total number of genes for respective functional annotation

Supplementary Table 3: Functional annotations of genes from Suit2-007 cells growing in lung environment

Categories[a]	Diseases or Functions Annotation[b]	p-Value	Predicted activation	Activation Z-score[c]	Number of genes[d]
Cell death & survival	Apoptosis	1,80E-16	de-creased	-2,144	LGALS4, IFI27, GSTA2, EDNRB, HOXA5, GSTA1, FGFR3,CRYAB, LGALS2, ID2, PPP1R1B, APP, LMO4, BDNF, DEFB,GDF15, AGT, EIF4G2, PRDX3, ARF4, TCP1, ITGB1, ASNS,EHF, SON, MX1, ATP1B3, CD24, ZFAND6, FOXO4, B2M,CTNNB1, UBA3, DPP4, NET1, PPM1A, BEX2, AQP1, IL1AANXA4, DKK1, DPEP1, IFI6, JUND, CDH1, FHL2, RPS6KA3,MSN, LGALS1, CSF2, KISS1, CLIP3, IGFBP6, TGM2, CXCL1,ANGPTL4, IGFBP3, EPHB6, CXCL8, CDCP1(59/175)
Cell death & survival	Cell viability of tumor cell lines	6,69E-07	increased	2,657	HBA1/HBA2, HOXA5, FGFR3, ID2, PPP1R1B, APP, BDNF, ARID1A, GDF15, TCP1, ITGB1, ASNS, CD24, CTNNB1, DPP4, PSMC4, BEX2, CDH1, RPS6KA3, DNAJB6, ARRB1, MDK, HSPA6, NFAT5, CSF2, TGM2, IGFBP3, CXCL8, IL1A (29/77)
Cellular growth, development, & proliferation	Cell proliferation of tumor cell lines	1,06E-13	increased	2,979	ADAM10,APP,CDH17,CXCL1,ERBB3,GABRP,GSK3B,IGFBP3,ILK,LGALS1,LOX,MYC,NAMPT,OGT,RRM2,STAT3,TMPO,TNF,WSB1(19/157)
Organismal development	Growth of organism	1,80E-04	increased	2,461	AMD1,APP,BMP4,CDC42,CNOT2,CRCP,CTNNB1,DEFB1,DICER1,DKK1,GSK3B,GSN,H19,IGFBP3,MID1IP1,PNPT1,SLC30A5,SUMO2,TNF (19/19)
Gene expression	Transacti-vation	8,91E-07	increased	2,607	APP, LMO4, BDNF, ARID1A, AGT, EHF, MX1, CTNNB1, DPP4, BEX2, ANXA4, THRA, CDH1, FHL2, NFIB, HERC5, ARRB1, LGALS1, CXCL8, IL1A (20/46)
Lipid metabo-lism &molec-ular transport	Release of fatty acid	1,54E-03	de-creased	-2,176	BDNF,CSF2,IL1A,IL32,KISS1,LEP,LGALS1,PTGS1,TNF (9/9)
Infectious diseases	Viral infection	1,40E-04	increased	2,953	HBA1/HBA2, CRYAB, DEFB1, ARID1A, IFIT1, PSENEN, TCN1,SPTBN1,ITGB1, CAV2, SON, MX1, CD24, PICALM, B2M, CTNNB1, DPP4, PSMC4, NUP153, ANXA2, EFNB2, SUMO2, DKK1, HCP5, HLA-B, RPS6KA3, LY6E, ARRB1, HSPA6, SLC2A3, LGALS1, CSF2, HNRNPU, ARL16, MAGT1, CLIP3, APOBEC3G, EPHB6, CXCL8, IL1A, IL23A (41/116)
Cellular movement	Invasion of melanoma cell lines	1,03E-03	de-creased	-2,157	BMP4,CDH1,CXCL8,FXYD5,ID2,MMP1,MX1,NFAT5,SDCBP,STAT3 (10/10)

[a] Categories are broader functions under which various gene cluster are annotated by Ingenuity pathway analysis.

[b]Diseases or functions annotations represent predicted biological functions of gene clusters derived Ingenuity Pathway Analysis.

[c]p-Value is the calculated probability derived from individual gene cluster in a defined annotation for the respective gene group.

[d]Predicted activation is the overall activation state (increased or decreased) of the functional annotation for respective genes in a given gene cluster.

[e]Activation Z-score is a defined quantity, which determines whether a biological function has significantly more “increased” predictions than “decreased” predictions (Z>0) or vice versa (Z<0).

[f]Number of genes represent selected genes based on significant modulation of expression versus the total number of genes for respective functional annotation.

Supplementary Table 4: RT-PCR experiment following siRNA knockdown of TGM2

TGM2-24 h	Cp 1	Cp 2	Cp 3	Average	Average GAPDH	Norma-lized	Average control	Cp difference.	Expression	% Kd.
**Control**	29.26	30.26	28.93	29.48	14.32	15.16	15.11			
**Control + Lipid**	29.57	29.52	28.8	29.30	14.23	15.06				
**1:4 , 50nM**		31.57		31.57	14.72	16.85		1.74	0.30	70
**1:5 , 50nM**	29.91	30.4	29.57	29.96	14.37	15.59		0.47	0.72	28
**1:4 , 100nM**	34.19	33.73	34.66	34.19	14.79	19.41		4.29	0.05	95
**TGM2-48 h**	**Cp 1**	**Cp 2**	**Cp 3**	**Average**	**Average GAPDH**	**Norma-lized**	**Average control**	**Cp difference**	**Expression**	**% Kd.**
**Control**	30.84	29.9	30.33	30.36	14.79	15.57	15.42			
**Control + Lipid**	30.96	29.79	29.5	30.08	14.81	15.28				
**1:4 , 50nM**		30.75	31.01	30.88	14.58	16.30		0.88	0.54	46
**1:5 , 50nM**	32.51	31.72	33.36	32.53	14.93	17.60		2.18	0.22	78
**1:4 , 100nM**	34.07		33.13	33.60	14.85	18.75		3.33	0.10	90

Table4 represent results of RT-PCR experiment following TGM2 knockdown in suit2-007 cells with different siRNA concentrations at 24 h and 48 h, respectively. Shown are the average Cp (crossing point) values for each triplicate treatment. The values for GAPDH (control) are also shown. The percent knockdown (% kd) of TGM2is shown in the last column.

Supplementary table5:

Selected genes and their respective proteins (Chip array experiment)

**Gene Name**	**Protein name**
ACP1	Adipocyte acid phosphatase) phosphatase 1
ACTN1	Alpha-actinin-1
ADAM8	Disintegrin and metalloproteinase domain-containing protein 8 (ADAM 8)
ADD3	Gamma-adducin (Adducin-like protein 70)
ADORA1	Adenosine receptor A1
AGR2	Anterior gradient protein 2 homolog (AG-2)
AGT	Angiotensinogen (Serpin A8)
AIF1	Allograft inflammatory factor 1 (AIF-1)
ANXA1	Annexin A1
ANXA2	Annexin A2 (Annexin II) (Annexin-2)
ANXA4	Annexin A4 (35-beta calcimedin) (Annexin IV)
APOBEC3G	DNA dC->dU-editing enzyme APOBEC-3G
APP	Amyloid-beta A4 protein (ABPP, APPI, APP)
AQP1	Aquaporin-1 (AQP-1)
AQP5	Aquaporin-5 (AQP-5)
ARF4	ADP-ribosylation factor 4
ARHGEF1	Rho guanine nucleotide exchange factor 1 (p115RhoGEF)
ARID1A	AT-rich interactive domain-containing protein 1A
ARL6IP1	ARL-6-interacting protein 1 (Aip-1), Apoptotic regulator in the membrane of the endoplasmic reticulum

ARPC3	Actin-related protein 2/3 complex subunit 3
ARRB1	Beta-arrestin-1 (Arrestin beta-1)
ATM	Serine-protein kinase ATM
ATP1B3	Sodium/potassium-transporting ATPase subunit beta-3
B2M	Beta-2-microglobulin
BCL2L1	Bcl-2-like protein 1 (Bcl2-L-1), Apoptosis regulator Bcl-X
BDNF	Brain-derived neurotrophic factor (BDNF), Abrineurin
BHLHE40	Class E basic helix-loop-helix protein 40 (bHLHe40)
BIRC2	Baculoviral IAP repeat-containing protein 2
BRE	BRISC and BRCA1-A complex member 2
CAPRIN2	Caprin-2 (C1q domain-containing protein 1)
CAV1	Caveolin1
CCL20	C-C motif chemokine 20 (Beta-chemokine exodus-1)
CCND3	G1/S-specific cyclin-D3
CD151	CD151 antigen (GP27), Membrane glycoprotein SFA-1
CD58	CD antigen CD58)
CD74	HLA class II histocompatibility antigen gamma chain (HLA-DR), CD antigen CD74)
CDC20	Cell division cycle protein 20 homolog (p55CDC)
CDC42	Cell division control protein 42 homolog
CDCP1	CUB domain-containing protein 1 (Membrane glycoprotein gp140)
CDH1	Cadherin-1 (CAM 120/80), Epithelial cadherin, E-cadherin
CDK2	Cyclin-dependent kinase 2
CDKN1A	Cyclin-dependent kinase inhibitor 1
CHMP5	Charged multivesicular body protein 5 (Chromatin-modifying protein 5)
CLDN2	Claudin-2 (SP82)
COL13A1	Collagen alpha-1(XIII) chain (COLXIIIA1)
CRYAB	Alpha-crystallin B chain (Alpha(B)-crystallin)
CSF2	Colony-stimulating factor) (CSF)
CSF2RA	Granulocyte-macrophage colony-stimulating factor receptor subunit alpha
CTGF	Connective tissue growth factor (CCN family member 2)
CTNNB1	Catenin beta-1 (Beta-catenin)
CXCL16	C-X-C motif chemokine 16

CXCL8	Interleukin-8 (IL-8)
DCN	Decorin (Bone proteoglycan II)
DDIT3	DNA damage-inducible transcript 3 protein (DDIT-3)
DEFB1	Beta-defensin 1 (BD-1), Defensin beta 1
DPP4	Dipeptidyl peptidase 4 (ADABP)
DYSF	Dysferlin (Dystrophy-associated fer-1-like protein)
EDNRB	Endothelin receptor type B (ET-B)
EHF	ETS homologous factor (hEHF)
EIF4G2	Eukaryotic translation initiation factor 4 gamma 2
ENG	Endoglin (CD antigen CD105)
EPCAM	Epithelial cell adhesion molecule (Ep-CAM)
EPHB6	Ephrin type-B receptor 6 (HEP)
ERBB3	Receptor tyrosine-protein kinase erbB-3
ERP29	Endoplasmic reticulum resident protein 29 (ERp29)
ETS1	Protein C-ets-1 (p54)
FBXO5	F-box only protein 5 (Early mitotic inhibitor 1)
FGFR3	Fibroblast growth factor receptor 3 (FGFR-3)
FGG	Fibrinogen gamma chain
FHL2	Four and a half LIM domains protein 2 (FHL-2)
FOS	Proto-oncogene c-Fos (Cellular oncogene fos)
FOSL1	Fos-related antigen 1 (FRA-1)
GADD45A	Growth arrest and DNA damage-inducible protein GADD45 alpha
GDF15	Growth/differentiation factor 15 (GDF-15)
GSK3B	Glycogen synthase kinase-3 beta (GSK-3 beta)
GSTA1	Glutathione S-transferase A1
GSTA2	Glutathione S-transferase A2
HAT1	Histone acetyltransferase 1
HERPUD1	Homocysteine-responsive endoplasmic reticulum-resident ubiquitin-like domain member 1 protein
HLA-A	HLA class I histocompatibility antigen,
HLA-DPA1	HLA class II histocompatibility antigen, DP alpha 1 chain
HLA-DRA	HLA class II histocompatibility antigen, DR alpha chain
HNRNPA1	Heterogeneous nuclear ribonucleoprotein A1 (hnRNP A1)
HNRPK	Heterogeneous nuclear ribonucleoprotein K (hnRNP K)
HOXA5	Homeobox protein Hox-A5

HYAL1	Hyaluronidase-1 (Hyal-1)
HYPK	Huntingtin-interacting protein K (Huntingtin yeast partner K)
ID2	DNA-binding protein inhibitor ID-2
IER3	Radiation-inducible immediate-early gene IEX-1
IFI27	Interferon alpha-inducible protein 27
IFI6	Interferon alpha-inducible protein 6
IFIT1	Interferon-induced protein with tetratricopeptide repeats 1 (IFIT-1)
IGF2	Insulin-like growth factor II (IGF-II)
IGFBP3	Insulin-like growth factor-binding protein 3 (IBP-3)
IL1A	Interleukin-1 alpha (IL-1 alpha)
IL23A	Interleukin-23 subunit alpha (IL-23 subunit alpha)
IL32	Interleukin-32 (IL-32) (Natural killer cells protein 4)
IL8	Interleukin-8 (IL-8) (C-X-C motif chemokine 8)
INF2	Inverted formin-2 (HBEBP2-binding protein C)
IRS1	Insulin receptor substrate 1 (IRS-1)
ITGB1	Integrin beta-1 (Fibronectin receptor subunit beta)
ITGB2	Integrin beta-2
JAK1	Tyrosine-protein kinase JAK1
JUN	Proto-oncogene c-Jun
JUND	Transcription factor jun-D
JUP	Junction plakoglobin (Catenin gamma)
KCNN4	Intermediate conductance calcium-activated potassium channel protein 4 (SK4)
KISS1	Metastasis-suppressor KiSS-1 (Kisspeptin-1)
KITLG	Kit ligand (Mast cell growth factor)
KLF9	Krueppel-like factor 9
KPNA2	Importin subunit alpha-1 (Karyopherin subunit alpha-2)
LAMA3	Laminin subunit alpha-3 (Epiligrin 170 kDa subunit)
LAMB3	Laminin subunit beta-3 (Epiligrin subunit bata)
LAMC2	Laminin subunit gamma-2 (Cell-scattering factor 140 kDa subunit)
LGALS1	Galectin-1 (Gal-1), (Beta-galactoside-binding lectin L-14-I)
LGALS2	Galectin-2 (Gal-2), Beta-galactoside-binding lectin L-14-II
LGALS4	Galectin-4 (Gal-4), Lactose-binding lectin 4
LMO4	LIM domain transcription factor LMO4
LTB	Lymphotoxin-beta (LT-beta)

LTBR	Lymphotoxin-beta receptor, Tumor necrosis factor C receptor
MAOA	Monoamine oxidase type A (MAO-A)
MAX	Protein max (Class D basic helix-loop-helix protein 4)
MDK	Midkine (MK), Amphiregulin-associated protein
MGAT3	Beta-1,4-mannosyl-glycoprotein 4-beta-N-acetylglucosaminyltransferase
MMP1	Matrix metalloproteinase-1
MSN	Moesin (Membrane-organizing extension spike protein)
MUC1	Mucin-1 (MUC-1)
MUC16	Mucin-16 (MUC-16)
MX1	Myxovirus resistance protein 1
MYC	Myc proto-oncogene protein
NAMPT	Nicotinamide phosphoribosyltransferase (NAmPRTase)
NCAPG2	Condensin-2 complex subunit G2
NCKAP1	Nck-associated protein 1 (NAP 1)
NDRG1	Protein NDRG1 (Differentiation-related gene 1 protein)
NFAT5	Nuclear factor of activated T-cells 5 (NF-AT5)
NFIB	Nuclear factor 1 B-type (NF1-B)
NR1H4	Bile acid receptor , (Nuclear receptor subfamily 1 group H member 4)
PDGFC	Platelet-derived growth factor C (PDGF-C)
PHLDA1	Pleckstrin homology-like domain family A member 1
PKM2	Pyruvate kinase PKM
PLAT	Tissue-type plasminogen activator (t-PA) chain B]
PLSCR1	Phospholipid scramblase 1 (PL scramblase 1)
PPARG	Peroxisome proliferator-activated receptor gamma
PPM1A	Protein phosphatase 1A (EC 3.1.3.16)
PRDX3	Thioredoxin-dependent peroxide reductase, mitochondrial
PRKCDBP	Protein kinase C delta-binding protein
PRNP	Alternative prion protein (AltPrP)
PRNP	Major prion protein (PrP) (ASCR)
PRODH	Proline dehydrogenase 1, mitochondrial
PTGS1	Prostaglandin G/H synthase 1
PTPN13	Tyrosine-protein phosphatase non-receptor type 13
PXN	Paxillin
RBBP4	Histone-binding protein RBBP4

RHOU	Rho-related GTP-binding protein RhoU
RPS6KA3	Ribosomal protein S6 kinase alpha-3
RRM2	Ribonucleoside-diphosphate reductase subunit M2
SAA1	Serum amyloid A-1 protein (SAA)
SDCBP	Syntenin-1 (Melanoma differentiation-associated protein 9)
SELP	P-selectin (CD62 antigen-like family member P)
SELP	Selenoprotein P (SeP)
SERPINA5	Plasma serine protease inhibitor (Serpin A5)
SERPINB5	Serpin B5 (Maspin)
SLC3A2	Solute carrier family 3 member 2
SLPI	Antileukoproteinase (ALP) (BLPI)
SOD2	Superoxide dismutase [Mn], mitochondrial
SON	Protein SON (Bax antagonist selected in saccharomyces 1)
SPDL1	Protein Spindly (hSpindly)
SPTLC1	Serine palmitoyltransferase 1
SSTR2	Somatostatin receptor type 2 (SS-2-R)
STAT3	Signal transducer and activator of transcription 3
SULF2	Extracellular sulfatase Sulf-2 (hSulf-2)
SVIL	Supervillin (Archvillin)
TCF2	Transcription factor 2, (TCF-2)
TCP1	T-complex protein 1 subunit alpha (TCP-1-alpha)
TGM2	Transglutaminase-2, (TGase-2)
THBS1	Thrombospondin-1
TMED10	Transmembrane emp24 domain-containing protein 10
TNF	Tumor necrosis factor (Cachectin), TNF-alpha
TNFAIP3	Tumor necrosis factor alpha-induced protein 3
TNFRSF1B	Tumor necrosis factor receptor superfamily member 1B
TNFRSF25	Tumor necrosis factor receptor superfamily member 25
TNFRSF6B	Tumor necrosis factor receptor superfamily member 6B
TNFSF10	Tumor necrosis factor ligand superfamily member 10
TPT1	Translationally-controlled tumor protein (TCTP)
TRIB3	Tribbles homolog 3 (TRB-3)
TRPV2	Transient receptor potential cation channel subfamily V member 2
UBA3	NEDD8-activating enzyme E1 catalytic subunit

VIPR1	Vasoactive intestinal polypeptide receptor 1 (VIP-R-1)
WSB1	WD repeat and SOCS box-containing protein 1
XAF1	XIAP-associated factor 1 (BIRC4-binding protein)
ZFAND6	AN1-type zinc finger protein 6
ZFP36	Zinc finger protein 823 (Zinc finger protein ZFP-36)
ZFP36	mRNA decay activator protein ZFP36

Table 5 contains the names of genes selected from chip array experiment. These genes were evaluated based on their functional annotations as discussed in result section.
